# RBMS3, a downstream target of AMPK, Exerts Inhibitory Effects on Invasion and Metastasis of Lung Cancer

**DOI:** 10.7150/jca.86572

**Published:** 2023-09-04

**Authors:** Shi-Lin Lv, Xu Zhou, Yuan-jun Li, Ling-yu Luo, De-Qiang Huang

**Affiliations:** 1 Hospital of Gastroenterology, Institute of Digestive Diseases, The First Affiliated Hospital of Nanchang University, Nanchang, China.; 2 Queen Mary university, Nanchang University, Nanchang 330006, Jiangxi Province, China.; 3 Collaborative Innovation Center for Diagnosis and Treatment of Digestive, Cardiovascular, and Neurological Diseases of Nanchang University, Nanchang, China.; 4 Department of Oncology, The First Affiliated Hospital of Nanchang University, Nanchang, China.

**Keywords:** AMPK, RBMS3, lung cancer, invasion and metastasis

## Abstract

**Background:** Lung cancer is a highly malignant disease, primarily due to its propensity for metastasis. AMP-activated protein kinase (AMPK), the principal downstream effector of Liver Kinase B1 (LKB1), orchestrates a broad spectrum of molecular targets, thereby constraining tumor invasion and metastasis. In parallel, the RNA-binding protein RBMS3 (RNA-binding motif, single-stranded-interacting protein 3) plays a pivotal role in the epithelial-mesenchymal transition (EMT), a pivotal process in tumorigenesis. Therefore, our research aims to clarify the important role of RBMS3 as a mediator in the LKB1/AMPK inhibition of tumor invasion and metastasis.

**Methods:** We investigated the expression and correlation between RBMS3 and LKB1 in lung cancer tissues utilizing immunohistochemistry and TCGA-LUAD data, respectively. The relationship between RBMS3 and clinical pathological features and prognosis of lung cancer was also analyzed. The functions of RBMS3 in lung cancer cell proliferation, invasion, and migration were investigated in real-time in *vitro*. Additionally, we investigated the effects of AMPK agonists and inhibitors to explore the mediating role of RBMS3 in AMPK-induced inhibition of lung cancer invasion and migration.

**Results:** The IHC and TCGA data both revealed low expression of RBMS3 in lung cancer. Moreover, we found that low expression of RBMS3 was positively associated with lung cancer's histological grade, clinical stage, and N stage. Additionally, low RBMS3 expression was associated with poor overall survival. Cox regression analysis revealed that RBMS3 was an independent prognostic factor for lung cancer patients. In *vitro* experiments verified that RBMS3 inhibited lung cancer cell proliferation, invasion, and migration. Furthermore, our findings suggested that RBMS3 played an essential role in mediating AMPK's inhibitory effect on lung cancer invasion and migration.

**Conclusion:** Our study highlights a novel mechanism by which LKB1/AMPK pathway activation inhibits lung cancer invasion and metastasis by promoting RBMS3 expression, offering insights in developing innovative lung cancer therapies.

## 1. Introduction

Lung cancer is the leading cause of cancer deaths worldwide. In recent years, both the incidence and mortality have increased significantly. According to the global cancer statistics, more than 2 million people are newly diagnosed with lung cancer every year 1, 2. Despite the markedly advanced therapeutic methods, most people still die from lung cancer, with metastasis accounting for most of the mortality 3, 4. Surgery is the first choice for treating cancer at early stage. However, most of patients are in advanced stage once diagnosed. With the development in biomarker monitoring, the five years survival of lung cancer patients has significantly increased 2, 5. Hence, unveiling the underlying mechanisms has become an escalating necessity to identify nascent therapeutic targets. This process may facilitate the blueprint for precision-driven and effective treatment modalities that help to improve the clinical prognosis of cancer patients.

Over the past decade, research on the LKB1/AMPK signaling pathway has unveiled its vital role in connecting cell metabolism and tumor suppression 6, 7. Liver Kinase B1 (LKB1), also known as Serine/Threonine Kinase 11 (STK11), is a conserved, ubiquitously expressed serine/threonine protein kinase with a tumor-suppressing role 8, 9. Remarkably, LKB1 inactivating mutations were found in around 30% of non-small cell lung cancer (NSCLC) cases 10. Loss of STK11/LKB1 function promotes tumorigenesis and progression by fostering angiogenesis, epithelial-mesenchymal transition (EMT) and cell polarity disruption 11. Adenosine MonoPhosphate-activated Protein Kinase (AMPK), the primary downstream target of LKB1, is a cellular energy sensor that is phosphorylated under energy stress 12. AMPK plays a pivotal role in inhibiting cancer migration and invasion. Low AMPK levels significantly enhance the glycolytic activity of lung cancer cells, inducing epithelial-mesenchymal transition and promoting invasion and migration 13. Moreover, low levels of AMPK are associated with poor prognosis in lung cancer patients 13. AMPK drives autophagy in renal cancer cells while suppressing their migration and invasion by impacting the downstream effector mTOR 14. It can also suppress TGF-β1-induced EMT and lower MMP-2 and MMP-9 levels in NSCLC by modulating its downstream target SIRT1 15. AMPK crucially mediates the antitumor functions of LKB1. To unravel the potential mechanisms, we need to identify and intensively research on new downstream targets that mediate AMPK's inhibition of tumor migration and invasion.

RNA-binding proteins are responsible for coordinating the post-transcriptional processes involved in the maturation, transport, and stability of various types of RNAs, with RNA binding motif, single-stranded-interacting protein 3 (RBMS3) being one of them 16, 17. RBMS3 has been found to play a crucial role in development, including embryonic pancreas, neural tube, and dorsal root ganglion 16, and its abnormal expression has been associated with the initiation and progression of various cancers 18-21. Research findings have illustrated the involvement of RBMS3 in the process of epithelial-mesenchymal transition (EMT), thereby suppressing distant metastasis of cancers. In breast cancer, RBMS3 exerts its inhibitory effect on cancer metastasis by modulating the expression of Twist1 22. In ovarian cancer, the deficiency of RBMS3 results in the activation of the Wnt/β-catenin pathway through downregulating its inhibitory proteins 20. In nasopharyngeal carcinoma, RBMS3 is capable of binding to the promoter region of c-Myc, an important downstream target in the Wnt/β-catenin pathway 23. However, the regulatory role of RBMS3 in the migration and invasion of lung cancer remains unclear.

In this study, we analyzed the expression of the RBMS3 in lung cancer patients and investigated the regulatory effect of LKB1/AMPK on RBMS3. We found that RBMS3 is downregulated in lung cancer tissues and is strongly associated with clinical malignant pathological features and poor prognosis of patients. Further research revealed that RBMS3 has an inhibitory effect on lung cancer cell proliferation and metastasis, suggesting its potential as a tumor suppressor gene. Additionally, we found that activation of the LKB1/AMPK axis promotes RBMS3 expression, thereby inhibiting lung cancer metastasis. Based on these results, we have revealed a novel mechanism that may provide a new direction for the treatment strategy of lung cancer, which warrants further investigation.

## 2. Methods and materials

### 2.1 Immunohistochemistry

Specimens were embedded in paraffin. Sections were spliced (4 μm), dewaxed, blocked, and incubated with primary antibody overnight at 4℃. Then the tissue sections were incubated with mouse anti-LKB1 (santa cruz, sc-32245, 1:100) and rabbit RBMS3 antibodies (Abcam, AB198248, 1:100) overnight at 4℃. After PBS washing, tissue sections were incubated by the secondary antibody labeled with horseradish peroxidase. Human Lung adenocarcinoma Tissue Microarray (ZL-LUC1601) was purchased from Shanghai Zhuoli Biotechnology Co. LTD. (Shanghai, China), and RBMS3 and LKB1 expression was measured by the automated VIS DIA VisioMorph system (Visiopamm®, HOERSHOLM, Denmark). The average H-score was used as the cut-off criterion and divided RBMS3 and LKB1 expression into high expression group and low expression group. The clinical and pathological information of the sample was obtained from the array manufacturer.

### 2.2 Bioinformatic analyses

We downloaded high-throughput sequencing RNA data from the TCGA database and Genotype-Tissue Expression project (GTEx). The R package "reshape2" was used to combine the expression matrix. The R packages "clusterProfiler" and "org.Hs.eg.db" were used to convert ENSG IDs from the Ensembl database to gene symbols, and the "limma" package was used for differential gene analysis. The "ggplot2" package was used for data visualization. Unpaired analysis of RBMS3 mRNA expression was performed in 33 cancer types, including LUAD. Paired analysis of RBMS3 expression was performed in LUAD patients, and their corresponding clinicopathologic information was also downloaded from the Lung Adenocarcinoma Project (LUAD) in TCGA database. Excluding patients with incomplete clinicopathological data, a total of 470 LUAD patients were included in the end. As the TCGA database is open to the public according to its guidelines, all written informed consent was obtained prior to data collection.

### 2.3 Cell culture, transfection and drug treatment

A549 and NCI-H1975 cell lines were maintained in RPMI-1640 supplemented with 10% fetal bovine serum (FBS) at 37°C in a 5% CO2 atmosphere. The A549-LKB1 and A549-LKB1-AMPK-KO cell lines were previously constructed in our laboratory 24. RBMS3 and LKB1 knockout plasmids were constructed according to the CRISPR-Cas9 system, and we designed small guide RNA sequences for RBMS3 and LKB1 (sgLKB1 1:sense 5'-tggactcggagacgctgtgc-3', antisense 5'-gcacagcgtctccgagtcca-3' sgLKB1 2:sense 5'-aggctcttacggcaaggtga-3', antisense 5'-tcaccttgccgtaagagcct-3' sgRBMS3:sense 5'-ttgctgtgcaaattcgctga-3', antisense 5'-tcagcgaatttgcacagcaa-3'). Then the annealed nucleotides were cloned into Esp3i-digested pLentiCRISPR V2 plasmid. After the vector was confirmed by sequencing, the 293T cell line was seeded and incubated for 24 hours before the transfection. Then plasmids were transfected into cells by Lipofectamine 3000 (Invitrogen, USA) in Opti-MEM medium (Invitrogen, USA). The empty V2 plasmids were used as control. Pax2 and Vsvg plasmid were added simultaneously for lentivirus package. The lentivirus was collected after 48 hours and then infects H1975 cells. The over-expression lentivirus of RBMS3 is acquired from the Genechem and then infects the A549 cell line. After lentivirus infection, all cells were incubated with 2 ug/ml puromycin for a week. Cells were incubated with metformin or phenformin or compound C or AIC at doses as indicated in the text.

### 2.4 RNA extraction, reverse transcription, and quantitative real-time PCR

All steps in RNA extraction should be performed on ice. Total RNA was extracted by TRIzol reagent (Transgen, China). Each group was quantified to 1g by NanoDrop 2000 (Thermo, USA) to standardize. The reverse transcription kit (Tiangen, China) was used for reverse transcription of RNA into cDNA according to its protocol. qRT-PCR was performed on ABI7500 (Bio-Rad, USA) using FastFire qPCR PreMix (Tiangen, China). GAPDH acts as a housekeeping gene to standardize mRNA from different cell lines. △△CT was used as the mathematical method for comparison between diverse groups. The primers in use are as follows:

GAPDH

Forward: ACGGGAAGCTCACTGGCATGG

Reverse: GGTCCACCACCCTGTTGCTGTA

RBMS3

Forward: GGGGAACAGTTGAGTAAAACCA

Reverse: ACAATTTTTCCATACGGTTGGCA

### 2.5 Western blotting analysis

Protein lysis buffer RIPA with proteinase inhibitor (PMSF) and phosphatase inhibitor (Thermo, USA) was applied to prevent protein dephosphorylation and degradation for 30 minutes on ice. The same concentration of protein in each group was obtained by BCA protein measuring kit (Solarbio, Beijing, China). Proteins were then separated on SDS-PAGE gel and transferred to NC membrane by semi-dry electroblotting for 10 minutes. 5% skim-milk was used to block membrane for following incubation. We incubated primary antibodies for 15h at 4℃ and the secondary antibody for 2h at room temperature. As RBMS3 expression is low in A549 cell line, its protein bands were visualized by Enhanced Chemiluminescence (ECL) solution (Thermo Scientific, USA) in ChemiDoc XRS system (Bio Rad, USA). The antibodies are listed following: Rabbit anti-human RBMS3 (Abcam, ab198248, 1:1000), Rabbit anti-human AMPK (Abcam, ab198248, 1:1000), Rabbit anti-human p-AMPK (Abcam, ab32047, 1:10000), Rabbit anti-human acetyl-CoA carboxylase (CST, 3676S, 1:1000), Rabbit anti-human p-ACC (CST, 11818S, 1:1000), Mouse anti-human Liver kinase B1 (santa cruz, sc-32245, 1:1000), Rabbit anti-human GAPDH (CST, 5174S, 1:1000) was used as a housekeeping gene.

### 2.6 Colony formation assay

The A549, A549-RBMS3, H1975 and H1975-RBMS3 KO cells were seeded into six-well plates and incubated for 14 days (1000 cell per well). Then the cells were fixed by 4% paraformaldehyde (Solarbio, China) and stained with 5% crystal violet solution both for 30 minutes. The colonies containing more than 50 cells were counted by Image J.

### 2.7 Cell proliferation assay

This assay was performed as described previously 25. In short, 2000 cells per 100μl culture medium were seeded in 96-well plate. Then 10 μl CCK8 reagent was added to the well after 24 and 48 hours. After culturing for 2 hours, the absorbance was measure by multi-mode microplate reader (Molecular Devices, United States) at 450nm wavelength.

### 2.8 Transwell assay

The assay was carried out as described previously 26. Briefly, we utilized 8-μm pore size Transwell chambers with or without Matrigel (BD Biosciences) to perform invasion and migration assays. Afterward, 4% paraformaldehyde was used for cell fixation followed by staining with 5% crystal violet for 30 minutes. Finally, the samples were observed under a microscope.

### 2.9 Scratch-wound assay

The cells were seeded into sterile 6-well plates and allowed to reach 80-100% confluency before creating a scratch using a sterile 10 μL pipette tip. Images of the cells were captured and analyzed under a microscope at 0 and 24 hours after scratch creation.

### 2.10 Statistical analysis

Statistical analyses were carried out using SPSS 11.5 software (SPSS Inc, Chicago, USA). The relationship between RBMS3 expression levels and clinicopathological parameters was assessed with the Chi-squared test and Wilcoxon rank sum test (for continuous variables). Survival curves were generated using Kaplan-Meier survival analysis. Univariate and multivariate survival analyses were performed using a Cox proportional hazards regression model. The correlation between RBMS3 and LKB1 protein expression levels was evaluated using Spearman's correlation coefficient. The ROC curve was established using the "pROC" package in R. A p-value of less than 0.05 was considered statistically significant.

## 3. Results

### 3.1 Expression of RBMS3 and LKB1 in Lung Cancer

**Figure 1:**
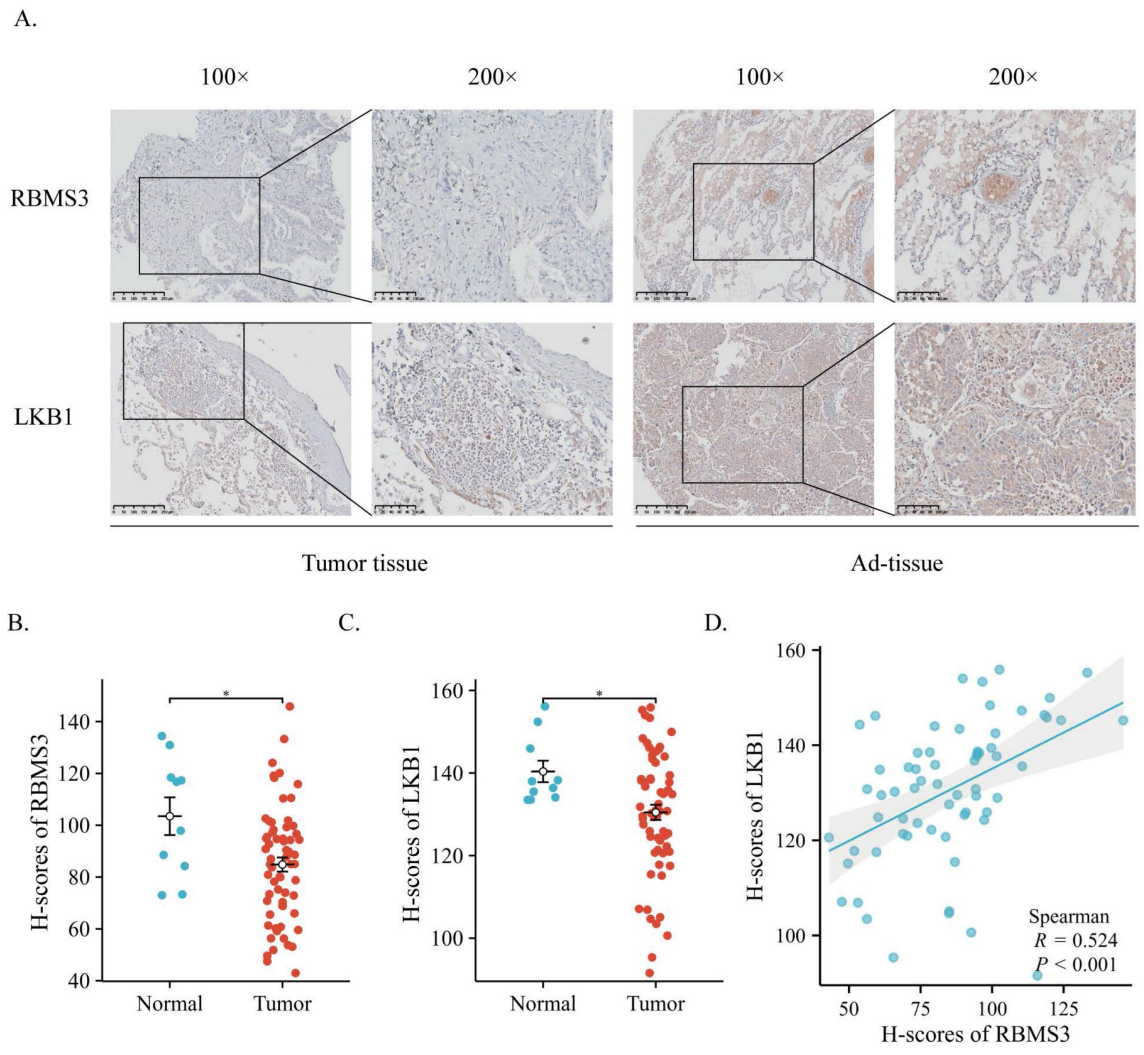
** Expression of RBMS3 and LKB1 in lung cancer via IHC.** (A) Representative images of RBMS3 and LKB1 staining in tumors and normal tissues. The right panel provides an enlarged view of the specific features highlighted in the rectangular image presented in the left panel. (Scale bar, 100 μm, 250 μm). Statistical analysis of the expression of RBMS3 (B) and LKB1 (C) proteins in lung cancer. (D) Correlation between RBMS3 and LKB1 in lung cancer patients. (*p<0.05).

To elucidate the potential relationship between RBMS3 and LKB1, we collected samples from 65 lung cancer patients and 10 adjacent normal lung tissue patients. IHC analysis was performed to assess the expression and localization of RBMS3 and LKB1 in lung cancer tissues and adjacent normal tissues (Figure 1A). The results revealed that RBMS3 and LKB1 were mainly localized in the cytoplasm. Statistical analysis showed significant decreases in the levels of RBMS3 (p<0.05) and LKB1 (p<0.05) in the lung cancer tissues compared to the adjacent normal tissues (Figure 1B, C). Moreover, there was a strong positive correlation between the expression of RBMS3 and LKB1 (R=0.524, P<0.001) (Figure 1D). Additionally, we investigated the clinicopathologic features of lung cancer patients with differential expression of RBMS3, as shown in Table 1. Our findings demonstrated that low expression of RBMS3 was significantly associated with Clinical N stage (p=0.026) and Pathological Grade (p=0.035).

### 3.2 Activation of LKB1/AMPK Axis Up-Regulates RBMS3 Expression in Lung Cancer

**Figure 2:**
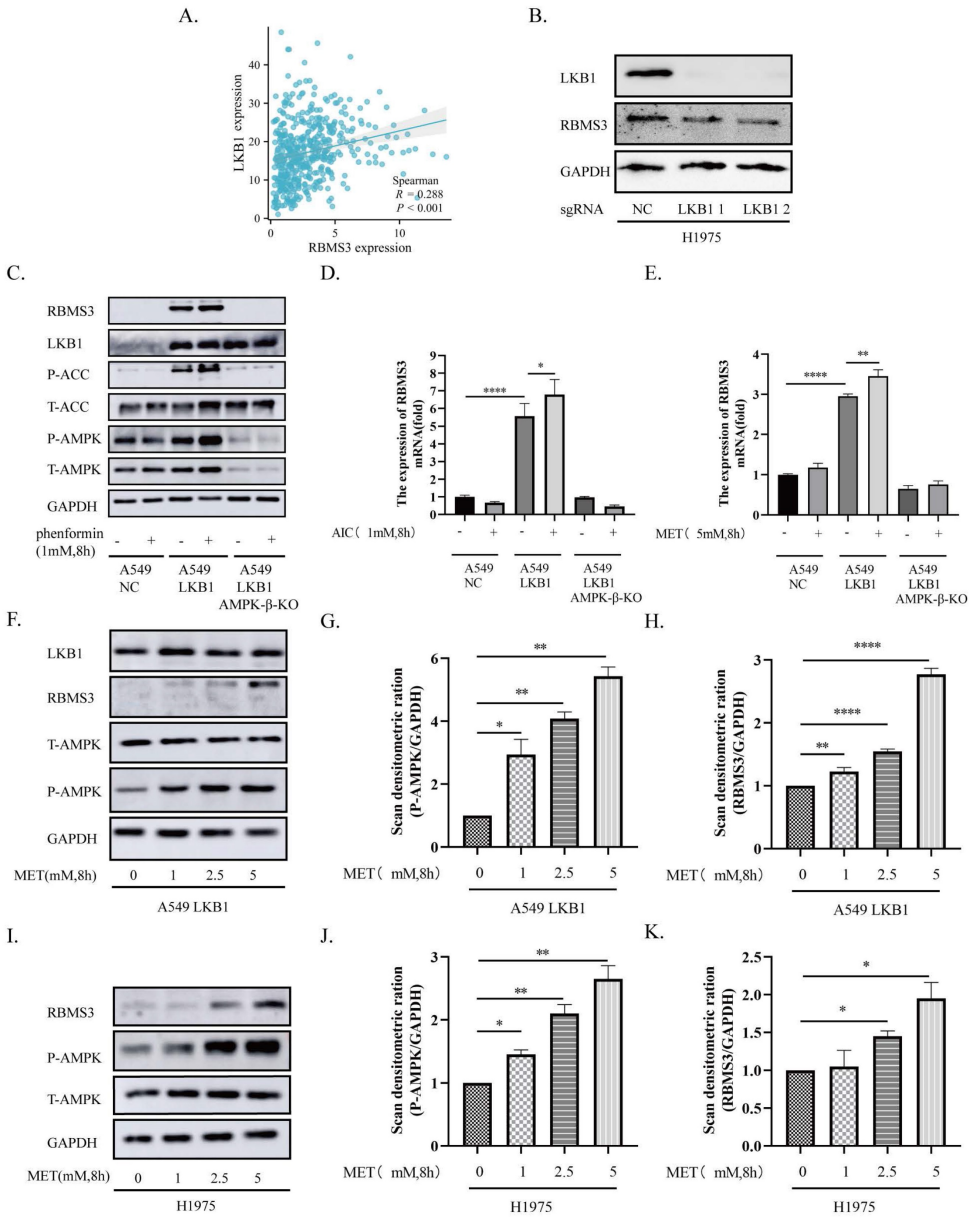
** The LKB1/AMPK axis promotes the expression of RBMS3.** (A) Correlation between RBMS3 and LKB1 in lung cancer from the TCGA database. (B) Western blotting to detect the change in RBMS3 expression after LKB1 knock-out. (C) A549-NC, A549-LKB1, A549-LKB1-AMPK-β-KO cells treated with phenformin (1mM) for 8 hours and cell extracts immunoblotted with antibodies as indicated. (D.E) A549-NC, A549-LKB1, A549-LKB1-AMPK-β-KO cells treated with AICAR (1mM) and metformin (5mM) for 8 hours. qPCR was used to detect RBMS3 expression. (F) A549-LKB1 and (I) H1975 cell lines were subjected to a gradient treatment of metformin for 8 hours, followed by immunoblotting analysis using the specified antibodies. The presented data represents a representative blot from three independent experiments. (G.H) The Western blots of (F) A549-LKB1 was scanned, and the resulting data was presented as densitometric unit ratios. (J.K) The Western blots of (I) H1975 was scanned, and the resulting data was presented as densitometric unit ratios. (*p<0.05, **p<0.01, ***p<0.001, ****p<0.0001).

As the IHC and TCGA dataset results showed that LKB1 and RBMS3 are positively related (Figure 1D, Figure 2A), we were curious to ask whether targeting LKB1/AMPK axis can impact the expression of RBMS3. Then we knocked out LKB1 in H1975 cells and observed a significant decrease in RBMS3 expression (Figure 2B). To investigate the relationship between LKB1 and RBMS3 at a functional level, we treated A549-NC, A549-LKB1, and A549-LKB1-AMPK-β-KO cell lines with the AMPK activator phenformin (1mM, 8h). The results showed a significant increase in the expression level of RBMS3 protein in A549-LKB1 cells compared to A549-NC cells, which was reversed upon knockout of the AMPK-β subunit. Interestingly, the addition of phenformin resulted in an increase in RBMS3 expression in A549-LKB1 cells, while no such observation was made in A549-NC cells (Figure 2C). To further validate this observation, we treated the cells with two other AMPK activators, AICAR (1mM, 8h) and metformin (5mM, 8h), and found that activation of the LKB1/AMPK axis was able to promote RBMS3 expression at the transcriptional level (Figure 2D, E).

We next sought to determine the effect of metformin, an AMPK activator, on the expression of RBMS3 and phosphorylation of T172 AMPK in A549-LKB1 and H1975 cells. Our results showed that metformin treatment led to increased T172 AMPK phosphorylation and RBMS3 expression in a dose-dependent manner (Figure 2F-K).

### 3.3 Downregulation of RBMS3 in Lung Cancer Revealed by Bioinformatics Analysis

**Figure 3:**
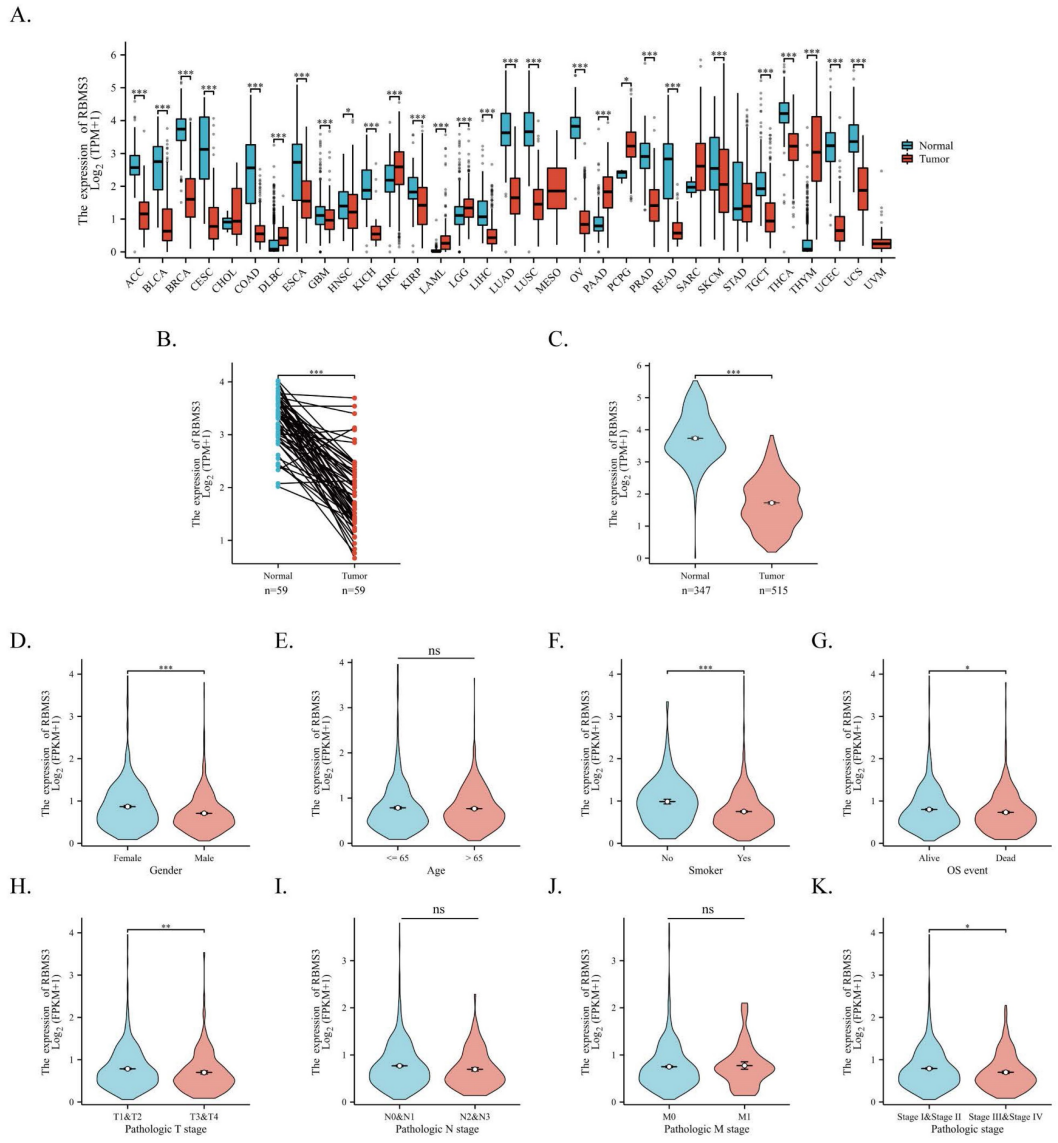
** Expression of RBMS3 in lung cancer and its subgroups.** (A) RBMS3 expression profiles across 33 types of cancers from the TCGA and GTEx databases. (B) Differences in RBMS3 expression within paired LUAD samples from the TCGA database. (C) Comparison of RBMS3 expression between cancerous and adjacent normal tissue in lung adenocarcinoma from the TCGA and GTEx databases. The Wilcoxon rank-sum test was used to assess the association between RBMS3 expression and various clinicopathological parameters, including gender (D), age (E), smoking history (F), mortality (G), clinical T stage (H), clinical N stage (I), clinical M stage (J), and clinical stage (K). (*p<0.05, **p<0.01, ***p<0.001, ****p<0.0001).

To elucidate the fundamental landscape of RBMS3 expression in cancer, we conducted a transcriptional analysis of RBMS3 in the TCGA and GTEx databases. Our results indicate that RBMS3 is predominantly downregulated in most cancers, including lung cancer (Figure 3A). Subsequently, we obtained 598 patient samples from the TCGA dataset and compared the expression of RBMS3 in lung adenocarcinoma with normal tissue. Our paired analysis revealed a significant decrease in RBMS3 expression in lung adenocarcinoma (Figure 3B). Furthermore, by integrating the TCGA and GTEx databases, we enlarged our sample size to 347 normal tissue samples to investigate RBMS3 expression and found a significant decrease in RBMS3 expression in LUAD (Figure 3C).

Additionally, we analyzed the RBMS3 expression levels in 1149 non-small cell lung cancer patients with various clinicopathological characteristics from TCGA. We observed a significant reduction in RBMS3 expression in males (Figure 3D), smokers (Figure 3F), patients who died (Figure 3G), T3 and T4 (Figure 3H), and patients with clinical stages III and IV (Figure 3K).

To further explore the clinical significance of RBMS3 in lung adenocarcinoma, we downloaded clinical and genetic expression data of 470 primary tumors from the TCGA database, which included patients' age, gender, grade, overall survival, stage, distant metastasis, survival status, T classification, and lymph nodes. Based on medium RBMS3 mRNA expression, we divided the samples into high or low groups. Our results showed that low RBMS3 mRNA expression was significantly associated with age (p=0.022), gender (P=0.002), clinical N stage (P=0.014), and clinical stage (P=0.001), respectively (Table 2).

### 3.4 Predictive Value of RBMS3 for Lung adenocarcinoma Diagnosis and Prognosis

**Figure 4:**
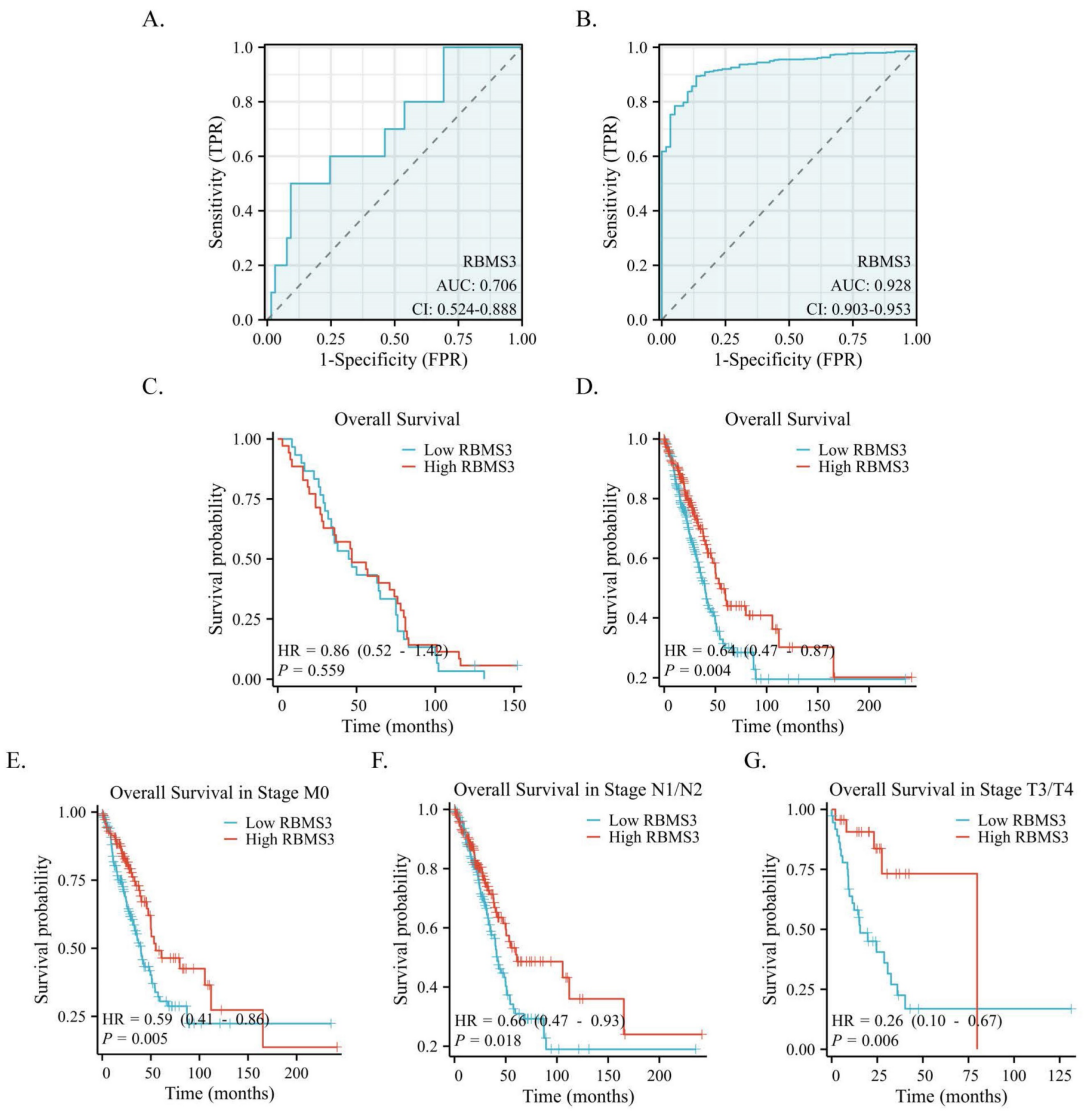
** Diagnostic and prognostic value of RBMS3 in lung cancer.** (A) ROC curves of RBMS3 protein expression in lung cancer tissue from hospitalized patients. (B) ROC curves of RBMS3 mRNA expression in lung cancer tissue cohort from the TCGA database. (C) Kaplan-Meier curves for overall survival in lung cancer for all cases from hospitalized patients. (D) Kaplan-Meier curves for overall survival in lung cancer for all cases from the TCGA database. (E) M0. (F) N1/N2. (G) T3/T4.

Based on IHC data, we applied a receiver operating characteristic (ROC) curve to assess the diagnostic value of RBMS3 in LUAD. Our findings revealed that the expression of RBMS3 can reliably predict the prognosis of lung cancer, with an AUC of 0.706 (Figure 4A). These results were further confirmed in a larger cohort of 470 lung cancer patients from the TCGA database, where the AUC of RBMS3 was 0.928 (Figure 4B). In addition, Kaplan-Meier survival analysis demonstrated that RBMS3 was not statistically significant in 65 LUAD patients (Figure 4C). As clinical samples were limited, we next investigated 470 samples in the TCGA database and found that low expression of RBMS3 mRNA predicts a poor prognosis in LUAD (P=0.004)(Figure 4D). Subgroup analysis showed that high expression of RBMS3 significantly affected the overall survival of M0 (P=0.005), N1/N2 (P=0.018), and T3/T4 (P=0.006) lung cancer patients, respectively (Figure 4E-G). Furthermore, univariate Cox regression analyses were performed to validate one another. As shown in Table 3, T stage, N stage, M stage, Clinical stage, and RBMS3 expression were associated with poor prognosis (p<0.05). Meanwhile, the results of multivariate Cox regression analysis showed that RBMS3 expression and T stage were independent prognostic factors in lung cancer patients (p<0.05).

### 3.5 RBMS3 inhibited the proliferation, migration, and invasion of lung cancer cells

**Figure 5:**
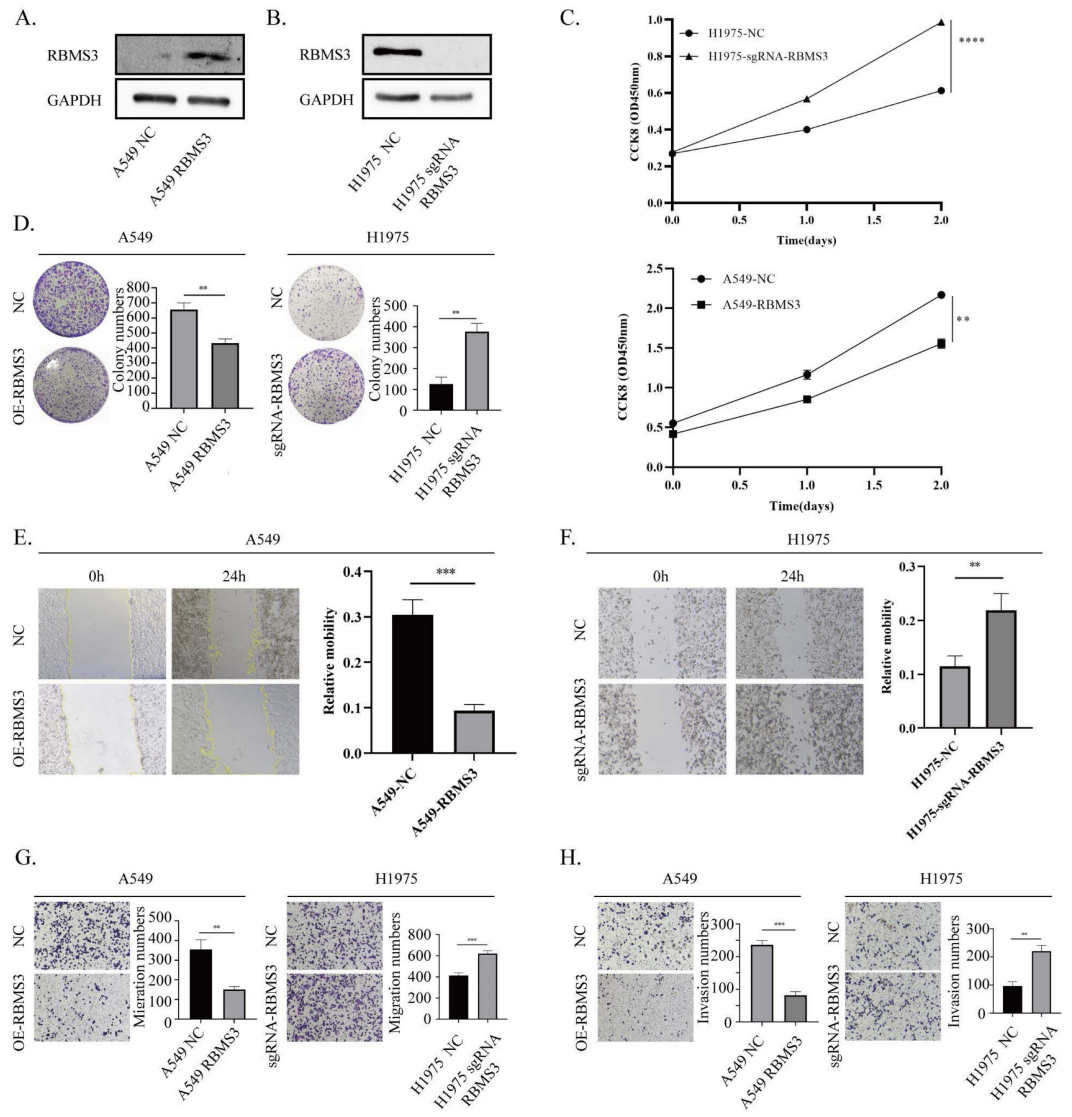
** RBMS3 suppresses lung cancer cell proliferation and metastasis.** (A) Up-regulation of RBMS3 expression in A549 cells. (B) Knockout of RBMS3 expression in H1975 cells. (C) CCK8 assay to evaluate the effect of RBMS3 on lung cancer cell proliferation. (D) Colony formation assay to investigate the effect of RBMS3 on lung cancer cell growth. (E.F) Scratch-wound assay to assess the effect of RBMS3 on lung cancer cell migration. (G.H) Transwell assay to test the effect of RBMS3 on cell migration and invasion ability. G) Migration assay, H) Invasion assay. (*p<0.05, **p<0.01, ***p<0.001, ****p<0.0001).

To investigate the biological function of RBMS3 in lung cancer, we conducted knockdown and overexpression experiments in NSCLC cell lines. Specifically, we knocked out RBMS3 in H1975 cells and overexpressed RBMS3 in A549 cells (Figure 5A, B), with empty vector-transfected cells serving as controls. We then performed cell growth assays, including CCK8 and colony formation assays, to assess the impact of RBMS3 on lung cancer cell proliferation. Our results showed that overexpression of RBMS3 significantly reduced the viability of A549 cells (p<0.01, Figure 5C), while knockdown of RBMS3 markedly increased the viability of H1975 cells (p<0.0001, Figure 5C). Consistent with these findings, colony formation assays revealed similar trends (Figure 5D).

To further explore the effects of RBMS3 on lung cancer cell migration and invasion, we conducted wound healing, Transwell migration, and invasion assays. Our results demonstrated that upregulation of RBMS3 decreased the migratory and invasive abilities of cells, as shown by wound healing and Transwell assays. Conversely, knockdown of RBMS3 increased the migratory and invasive abilities of cells (Figure 5E-H). Taken together, these findings suggest that RBMS3 plays a critical role in NSCLC progression.

### 3.6 RBMS3 was essential for AMPK Activation-Mediated Suppression of Lung Cancer Invasion and Migration

**Figure 6:**
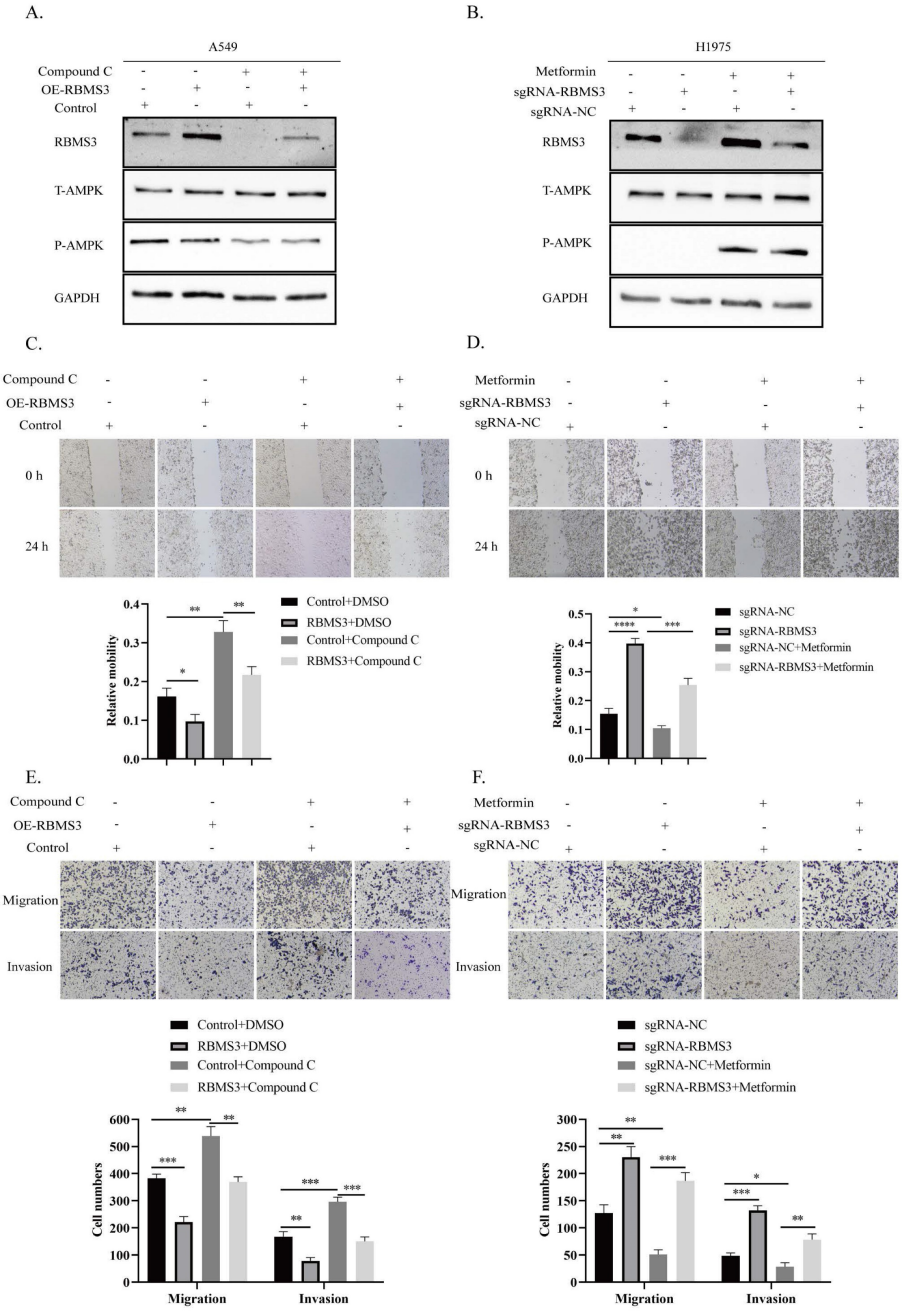
** RBMS3 mediates the function of AMPK in inhibiting lung cancer cell invasion and metastasis.** (A) Western blot analysis of RBMS3 expression in A549 cells co-transfected with RBMS3 overexpression plasmid, compound C, and corresponding controls. (B) Western blot analysis of RBMS3 expression in H1975 cells co-transfected with sgRNA-RBMS3, Metformin, and corresponding controls. (C, D) Cell scratch assays demonstrated that RBMS3 inhibited lung cancer cell migration and that RBMS3 mediated the function of AMPK in lung cancer cell migration. (E, F) Transwell migration and invasion assays revealed that RBMS3 inhibited the migration and invasion of lung cancer cells, and that RBMS3 mediated the effect of AMPK on the migration and invasion of lung cancer cells. (*p<0.05, **p<0.01, ***p<0.001, ****p<0.0001).

Previous results have demonstrated that AMPK activation, induced by metformin, can promote RBMS3 expression. In this study, we aimed to investigate whether RBMS3 is essential for AMPK-mediated suppression of lung cancer migration and invasion. To test this hypothesis, we utilized the AMPK inhibitor, compound C, to suppress RBMS3 expression in A549 cells, while RBMS3 overexpression plasmids partially counteracted the inhibitory effect of compound C on RBMS3 expression (Figure 6A). Conversely, sgRNA-RBMS3 partially reversed the promoting effect of metformin, an AMPK activator, on RBMS3 expression (Figure 6B). As expected, RBMS3 overexpression plasmids partially counteracted the promoting effect of compound C on A549 cell migration and invasion (Figure 6C, E), while sgRNA-RBMS3 partially reversed the inhibitory effect of metformin on H1975 cell migration and invasion (Figure 6D, F). Our findings indicate that RBMS3 functions as a tumor suppressor in lung cancer cells and acts as a mediator of AMPK signaling in these cells.

## 4. Discussion

It is widely recognized that mutations in key genes can trigger tumorigenesis and confer invasive properties to cancer cells. Notably, STK11/LKB1 has been found to be dysregulated in various types of tumors 27, 28, with lung adenocarcinoma exhibiting a higher incidence of mutations 29. These mutations frequently co-occur with KRAS mutations and are associated with reduced overall survival 30. AMPK, a vital downstream effector of the tumor suppressor LKB1, is widely regarded as a metabolic tumor suppressor due to its pivotal role in energy metabolism 31. Therefore, targeting the LKB1/AMPK pathway represents a promising anticancer strategy.

Our investigation reveals that the expression of LKB1 is significantly reduced in lung cancer tissues compared to normal tissues, corroborating previous studies 32. Additionally, studies have reported that the expression level of AMPK in lung cancer cells is also significantly lower than in normal bronchial epithelial cells, and knocking out AMPK induces the EMT process and enhances invasion and migration 13, 33, which is consistent with our findings. we demonstrated that AMPK activation exerts inhibitory effects on the invasion and migration of lung cancer cells, as evidenced by treatment with AMPK activators and inhibitors. Notably, our data revealed that RBMS3 protein expression levels increase upon AMPK activation. IHC and TCGA data analyses revealed a significant positive correlation between LKB1 and RBMS3 expression, suggesting that RBMS3 may be a downstream target of LKB1/AMPK signaling.

RBMS3, an RNA-binding protein located in the human chromosome 3p23-p24 region, is frequently deleted or mutated in cancer. As a member of the c-Myc single-strand binding protein family, RBMS3 plays a crucial role in DNA replication, transcription, apoptosis induction, and cell cycle progression 34, 35. Recent studies have demonstrated that RBMS3 is aberrantly expressed in various cancers, including breast cancer 36, ovarian cancer 37, and prostate cancer 38. Moreover, RBMS3 has been identified as a core transcription factor that regulates lung adenocarcinoma-related genes 39. However, the precise expression and function of RBMS3 in lung cancer remain unclear. To address this issue, we conducted IHC to measure the expression of RBMS3 in 75 lung cancer tissues and normal lung tissues. Our results showed that RBMS3 was significantly downregulated in lung cancer tissues. To validate these findings, we also analyzed data from the TCGA database, which consistently demonstrated lower expression of RBMS3 in lung cancer tissues. Clinically, we found that the expression of RBMS3 was strongly associated with the histological grade, clinical stage, and N stage of lung cancer. Furthermore, prognosis analysis based on the TCGA database revealed that lung cancer patients with low expression of RBMS3 had poorer survival, and RBMS3 was an independent prognostic factor for lung cancer. Taken together, our findings suggest that RBMS3 may serve as a promising tumor marker and prognostic indicator for lung cancer.

Research has unveiled RBMS3 as a novel potential regulator of epithelial-mesenchymal transition (EMT). In breast cancer, RBMS3 plays a pivotal role in maintaining the mesenchymal phenotype, invasiveness, and migratory ability. RBMS3 exerts its anti-EMT effect by modulating the expression and stability of PRRX1 19. Moreover, in gastric cancer, RBMS3 overexpression significantly reduces the invasive ability of cancer cells 16. To elucidate the precise function and mechanism of RBMS3 in lung cancer, we conducted a series of in vitro experiments. Our findings demonstrate that RBMS3 acts as a tumor suppressor gene in lung cancer, suppressing the migration, invasion, and proliferation of tumor cells. However, the exact mechanism underlying RBMS3 regulation in cancer remains to be fully elucidated. Previous studies have suggested that the long non-coding RNA MEG3 upregulates RBMS3 expression, thereby inhibiting breast cancer proliferation and apoptosis 36. Additionally, AMPK is implicated in the occurrence and progression of various cancers 40. Our results shed light on another mechanism for regulating RBMS3 expression. Specifically, we found that in lung cancer cells, LKB1 positively regulates RBMS3 expression, and this effect is abolished upon AMPK knockout. Furthermore, AMPK activation induced by metformin promotes RBMS3 expression and inhibits cancer cell invasion and migration in an RBMS3-dependent manner. Therefore, we propose that AMPK activation may represent another mechanism for promoting RBMS3 expression. Currently, the regulatory mechanism through which AMPK modulates RBMS3 expression remains elusive. Increasing evidence indicates that long non-coding RNAs (lncRNAs) can wield regulatory control over the expression and function of DNA, RNA, and proteins by virtue of their interplay, thereby wielding a profound impact on tumor onset and progression 41, 42. It is conceivable that AMPK may exert its regulatory influence indirectly through the modulation of an as-yet uncharacterized lncRNA. Consequently, our forthcoming investigations will center on the identification of the precise lncRNA responsible for mediating this effect.

In summary, the downregulation of RBMS3 expression holds significant prognostic implications for patients with lung cancer. Our study has further uncovered a novel mechanism for the inhibition of lung cancer progression, whereby the activation of RBMS3 is mediated by AMPK activation. This RBMS3-dependent mechanism sheds light on a promising therapeutic strategy for the treatment of lung cancer.

## Figures and Tables

**Table 1:** Correlation between RBMS3 Expression and Clinical Pathological Features of Lung Cancer by IHC

Characteristics	Low expression of RBMS3	High expression of RBMS3	P
n	30	35	
Age, n (%)			0.229
<65	23 (76.7%)	22 (62.9%)	
≥65	7 (23.3%)	13 (37.1%)	
Gender, n (%)			0.070
Male	8 (26.7%)	17 (48.6%)	
Female	22 (73.3%)	18 (51.4%)	
Clinical T stage, n (%)			0.829
T1-T2	26 (86.7%)	32 (91.4%)	
T3-T4	4 (13.3%)	3 (8.6%)	
Clinical N stage, n (%)			0.026
N0-N1	21 (70%)	32 (91.4%)	
N2-N3	9 (30%)	3 (8.6%)	
Clinical M stage, n (%)			0.638
M0	27 (90%)	29 (82.9%)	
M1	3 (10%)	6 (17.1%)	
Clinical stage, n (%)			0.078
I-II	16 (53.3%)	26 (74.3%)	
III-IV	14 (46.7%)	9 (25.7%)	
Pathological Grade, n (%)			0.035
I-II	11 (36.7%)	22 (62.9%)	
III-IV	19 (63.3%)	13 (37.1%)	

**Table 2:** Correlation between RBMS3 Expression and Clinical Pathological Features of Lung Cancer in TCGA Database

Characteristics	Low expression of RBMS3	High expression of RBMS3	P
n	235	235	
Age, n (%)			0.022
<65	112 (50.2%)	90 (39.5%)	
≥65	111 (49.8%)	138 (60.5%)	
Gender, n (%)			0.002
Male	124 (52.8%)	91 (38.7%)	
Female	111 (47.2%)	144 (61.3%)	
Clinical T stage, n (%)			0.059
T1-T2	196 (83.8%)	209 (89.7%)	
T3-T4	38 (16.2%)	24 (10.3%)	
Clinical N stage, n (%)			0.014
N0-N1	186 (80.2%)	201 (88.5%)	
N2-N3	46 (19.8%)	26 (11.5%)	
Clinical M stage, n (%)			0.105
M0	163 (91.1%)	150 (95.5%)	
M1	16 (8.9%)	7 (4.5%)	
Clinical stage, n (%)			0.001
I-II	167 (72%)	195 (84.4%)	
III-IV	65 (28%)	15.6%)	

**Table 3:** Cox Regression Analysis of Clinical Prognosis in Lung Cancer Patients

Characteristics	HR (95% CI) Univariate analysis	P value Univariate analysis	HR (95% CI) Multivariate analysis	P value Multivariate analysis
Age (≥65 vs. <65 years)	0.852 (0.627 - 1.158)	0.307		
Sex (male vs. female)	0.865 (0.641 - 1.168)	0.343		
Clinical T stage (T3-T4 vs. T1-T2)	2.092 (1.405 - 3.115)	< 0.001	1.874 (1.149 - 3.056)	0.012
Clinical N stage (N2-N3 vs. N0-N1)	2.009 (1.387 - 2.910)	< 0.001	1.790 (0.817 - 3.924)	0.146
Clinical M stage (M1 vs. M0)	1.802 (1.013 - 3.203)	0.045	1.361 (0.590 - 3.140)	0.470
Clinical stage (stage III-IV vs. stage I-II)	2.146 (1.549 - 2.973)	< 0.001	1.137 (0.493 - 2.618)	0.764
RBMS3 (High vs. low)	0.642 (0.473 - 0.871)	0.004	0.665 (0.463 - 0.955)	0.027

## Abbreviations

RBMS3: RNA-binding motif, single-stranded-interacting protein 3
p-AMPK: phospho-AMP-activated protein kinase
AMPK: Adenosine MonoPhosphate-activated Protein Kinase
AUC: area under the curve
ROC: Receiver operating characteristic
TCGA: The Cancer Genome Atlas
IHC: immunohistochemical
Ad-tissue: adjacent normal tissue
EMT: Epithelial-Mesenchymal Transition
OS: overall survival
